# Effects of Periconceptional Multivitamin Supplementation on Folate and Homocysteine Levels Depending on Genetic Variants of Methyltetrahydrofolate Reductase in Infertile Japanese Women

**DOI:** 10.3390/nu13041381

**Published:** 2021-04-20

**Authors:** Keiji Kuroda, Takashi Horikawa, Yoko Gekka, Azusa Moriyama, Kazuki Nakao, Hiroyasu Juen, Satoru Takamizawa, Yuko Ojiro, Koji Nakagawa, Rikikazu Sugiyama

**Affiliations:** 1 Center for Reproductive Medicine and Implantation Research, Sugiyama Clinic Shinjuku, Tokyo 116-0023, Japan; hori@sugiyama.or.jp (T.H.); 405fmv@gmail.com (Y.G.); azt0219@gmail.com (A.M.); kazuki.nakao@gmail.com (K.N.); juen@home.email.ne.jp (H.J.); takami@sugiyama.or.jp (S.T.); ojiro@sugiyama.or.jp (Y.O.); nakagawa-jiko@spice.ocn.ne.jp (K.N.); riki@sugiyama.or.jp (R.S.); 2 Department of Obstetrics and Gynecology, Faculty of Medicine, Juntendo University, Tokyo 113-8421, Japan

**Keywords:** folic acid, homocysteine, infertility, methyltetrahydrofolate reductase (MTHFR), multivitamin supplementation, Vitamin D

## Abstract

Methylenetetrahydrofolate reductase (MTHFR) has various polymorphisms, and the effects of periconceptional folic acid supplementation for decreasing neural tube defects (NTDs) risk differ depending on the genotypes. This study analyzed the effectiveness of multivitamin supplementation on folate insufficiency and hyperhomocysteinemia, depending on MTHFR polymorphisms. Of 205 women, 72 (35.1%), 100 (48.8%) and 33 (16.1%) had MTHFR CC, CT and TT, respectively. Serum folate and homocysteine levels in women with homozygous mutant TT were significantly lower and higher, respectively, than those in women with CC and CT. In 54 women (26.3% of all women) with a risk of NTDs, multivitamin supplementation containing folic acid and vitamin D for one month increased folate level (5.8 ± 0.9 to 19.2 ± 4.0 ng/mL, *p* < 0.0001) and decreased the homocysteine level (8.2 ± 3.1 to 5.8 ± 0.8 nmol/mL, *p* < 0.0001) to minimize the risk of NTDs in all women, regardless of MTHFR genotype. Regardless of MTHFR genotype, multivitamin supplements could control folate and homocysteine levels. Tests for folate and homocysteine levels and optimal multivitamin supplementation in women with risk of NTDs one month or more before pregnancy should be recommended to women who are planning a pregnancy.

## 1. Introduction

Neural tube defects (NTDs), such as spina bifida and anencephaly, are the most common congenital malformations in the process of neurological development, and annually, 300,000 to 400,000 infants with NTDs are born [1,2]. Periconceptional folate consumption can prevent approximately half of NTD cases [3,4,5,6], yet folic acid cannot be synthesized in the body. Therefore, daily folic acid supplementation is recommended in women who desire conception [7]. In most developed countries, the prevalence of spina bifida has decreased; however, it still has not diminished in Japan [8,9].

**Figure 1:**
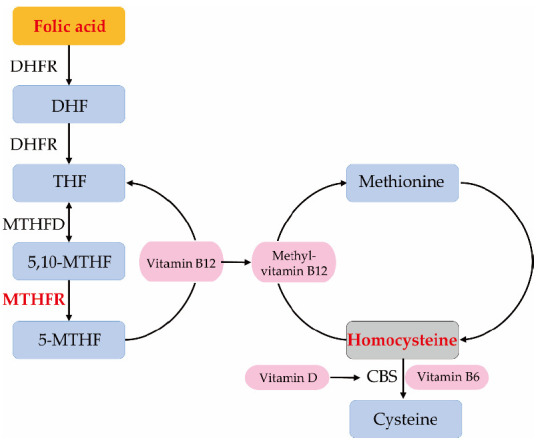
Folic acid and homocysteine metabolic pathway. Folic acid is enzymatically converted to tetrahydrofolate (THF) by dihydrofolate reductase (DHFR) via dihydrofolate (DHF). THF is converted to 5,10-methylenetetrahydrofolate (5,10-MTHF) by methylenetetrahydrofolate dehydrogenase (MTHFD) and catalyzed to 5-methyltetrahydrofolate (5-MTHF) by methylenetetrahydrofolate reductase (MTHFR). 5-MTHF can be converted to THF again when a methyl group is passed to vitamin B12, resulting in methyl-vitamin B12. The methyl group can metabolize homocysteine into methionine. Homocysteine is also catabolized to cysteine by a vitamin B6-dependent enzyme, cystathionine β-synthase (CBS). Vitamin D has the effect of enhancing enzyme activity of CBS.

Folic acid is enzymatically reduced and converted to tetrahydrofolate (THF) by dihydrofolate reductase via dihydrofolate. THF is converted to 5,10-methylenetetrahydrofolate (5,10-MTHF) by methylenetetrahydrofolate dehydrogenase and catalyzed to 5-methyltetrahydrofolate (5-MTHF) by methylenetetrahydrofolate reductase (MTHFR). 5-MTHF can be converted to THF again when a methyl group is passed to vitamin B12, resulting in methyl-vitamin B12. The methyl group from methyl-vitamin B12 can metabolize a cytotoxic molecule, homocysteine, into methionine. Homocysteine is also catabolized to cysteine by a vitamin B6-dependent enzyme, cystathionine β-synthase (CBS) [10] (Figure 1). Impaired metabolism of homocysteine is directly involved in increased incidence of NTDs; therefore, homocysteine levels are increased by the insufficiency of either vitamin B6, B12 or folic acid [11]. Daly et al. reported that folate levels were dose-dependently correlated with incidence risks of NTDs [12]. The threshold of red blood cell folate levels for minimizing the risk of NTDs of 906 nmol/L, equivalent to plasma folate levels of 7.0 ng/mL, has been established [12]. In particular, women with serum folate levels of <5.0 ng/mL have significantly increased NTD risk. Furthermore, MTHFR polymorphism, such as the point mutation of nucleotide 677, is also associated with decreased enzymatic activity, leading to increased homocysteine levels [13] and the risk of NTD development [14,15].

Recently, the relationship between vitamin D insufficiency or deficiency and homocysteine level elevation has also been reported [16,17]. Vitamin D supplementation can decrease the total homocysteine levels and reduce the risk of cardiovascular diseases with hyperhomocysteinemia [18,19]. Furthermore, in recurrent pregnancy losses (RPL), women with homozygous mutation of MTHFR C677T (677TT) have lower vitamin D and higher homocysteine levels compared with those with MTHFR 677CC and 677CT [16]. Vitamin D has the effect of enhancing the enzyme activity of CBS [20]; therefore, vitamin D is a key factor in controlling homocysteine levels and decreasing NTD risk.

Generally, multivitamin supplementation, including folic acid and vitamins B6, B12 and D in women with NTD risk is important as preconception care, and its importance needs to be widely disseminated to reduce the incidence of NTDs in Japan. However, the rates of folate insufficiency, hyperhomocysteinemia and MTHFR gene polymorphisms have not been examined in infertile women in Japan. Moreover, MTHFR polymorphisms expectedly influence the therapeutic effects of multivitamin supplementation; however, the methods of optimal supplementation, depending on MTHFR genotypes, remain unknown. In addition, multiple-micronutrient supplementation including vitamins and minerals has potential benefits on female fecundity with antioxidant effects [21]. Therefore, multivitamin supplementation may improve pregnancy outcomes in fertility treatment. In this study, we analyzed the effectiveness of multivitamin supplementation on folate insufficiency and homocysteine level elevation, depending on MTHFR polymorphisms and examined the rates of pregnancy and miscarriage in fertility treatment by the MTHFR genotype following supplementation.

## 2. Materials and Methods

### 2.1. Patient Selection

**Figure 2:**
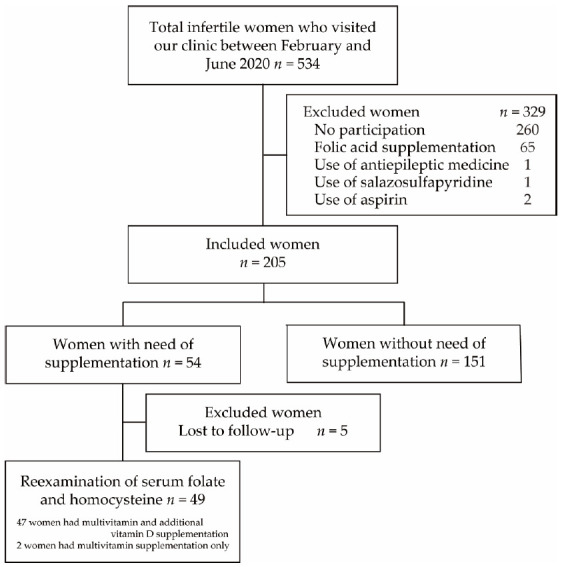
Flowchart of patient selection. Of the 534 consecutive infertile Japanese women who visited our clinic between February and June 2020, 205 were recruited after excluding 329 who refused participation (*n* = 260) and had folic acid supplementation (*n* = 65) and/or drugs that potentially inhibit folate absorption or conversion to its active form, including antiepileptic medications (*n* = 3) and anti-inflammatory drugs, such as sulfasalazine (*n* = 1) and aspirin (*n* = 2). Of the recruited women, 54 with serum folate levels of <7.0 ng/mL and/or hyperhomocysteinemia with homocysteine levels of >13.5 nmol/mL received daily multivitamin supplementation. After excluding five women without follow-up, 49 underwent remeasurement of folate and homocysteine levels after one month.

This study was approved by the local ethics committee of Sugiyama Clinic (No. 19-003; Tokyo, Japan). Blood and oral mucosa samples were collected after obtaining written informed consent. Of the 534 consecutive infertile Japanese women aged ≤42 years who visited our clinic between February and June 2020 for fertility treatment, 205 women were recruited after excluding 329 who refused participation (*n* = 260) and had folic acid supplementation (*n* = 65) and/or drugs that potentially inhibit folate absorption or conversion to its active form, including antiepileptic medications (*n* = 3) [22] and anti-inflammatory drugs, such as sulfasalazine (*n* = 1) and aspirin (*n* = 2) [22,23] (Figure 2). Infertility was diagnosed as the failure to achieve clinical pregnancy after 12 months or more of unprotect intercourse.

### 2.2. Multivitamin Prospective Intervention Study

Before the start of the study, blood and oral mucosa samples were obtained for the analysis of the serum levels of the folate, homocysteine, and vitamin D and MTHFR genotypes. Women with serum folate levels of <7.0 ng/mL have an increased risk of NTDs [12]. Therefore, of the 205 recruited women, 54 had folate levels of <7.0 ng/mL and/or hyperhomocysteinemia with homocysteine levels of >13.5 nmol/mL. After excluding five women without follow-up, 49 received daily multivitamin supplementation (Elevit^®^, Bayer Yakuhin, Ltd., Tokyo, Japan) and underwent the remeasurement of serum folate and homocysteine levels every month (28 to 35 days) until achieving folate level of ≥7.0 ng/mL and homocysteine level of <13.5 nmol/mL (Figure 2). Multivitamin supplementation contains 800 μg of folic acid, 1.3 mg of vitamin B1, 1.5 mg of vitamin B2, 12 mg of niacin, 1.4 mg of vitamin B6, 2.8 μg of vitamin B12, 5.0 mg of pantothenic acid, 50 μg of biotin, 100 mg of vitamin C, 7200 μg of β-carotene, 7.0 μg of vitamin D, 6.5 mg of vitamin E, 125 mg of calcium, 100 mg of magnesium, 21.5 mg of iron, 7.5 mg of zinc, 0.9 mg of copper, and 1.0 mg of manganese. Vitamin D insufficiency with storage-type vitamin D 25-hydroxyvitamin D3 (25 OHVD) levels of <30 ng/mL was also treated with vitamin D supplementation (Sugiyama Clinic Original Vitamin D supplement, Calinesse, Tokyo, Japan) containing vitamin D3 (cholecalciferol). Additional vitamin D supplementation was provided at a daily dose of 25 or 50 μg (32 or 57 μg in total) in infertile patients with 25 OHVD levels of ≥20 and <30 ng/mL or <20 ng/mL, respectively, according to our previous study [24,25]. Of 49 who reexamined serum folate and homocysteine levels, 47 women had additional vitamin D supplementation. Changes in the serum folate and homocysteine status were analyzed in patients with CC, CT and TT genotypes of MTHFR C677T.

**Figure 3:**
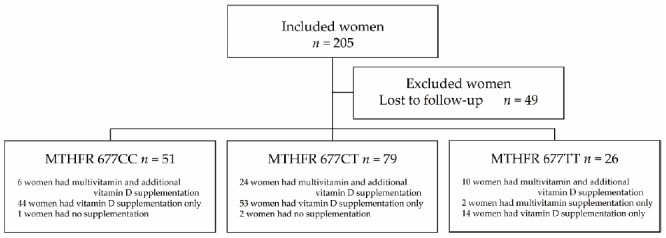
Flowchart of pregnancy outcomes. Of the 205 recruited women, we examined pregnancy outcomes in 51, 79 and 26 women with MTHFR 677CC, CT and TT genotypes, respectively, after excluding 49 without follow-up. Pregnancy outcomes within six months in three MTHFR genotypes were compared.

We also examined the pregnancy outcomes in three MTHFR genotypes as a secondary outcome. Of the 205 recruited women, 49 did not undergo fertility treatment because of the COVID-19 pandemic. Therefore, we compared the pregnancy outcomes within six months in 51, 79 and 26 women with MTHFR 677CC, CT and TT genotypes, respectively (Figure 3). A clinical pregnancy was defined when an intrauterine gestational sac was detected by transvaginal ultrasound. A miscarriage was defined as the loss of clinical pregnancy.

### 2.3. Measurement of Serum Folate, Homocysteine and Vitamin D Levels and MTHFR Genotypes

The obtained blood samples were sent to SRL Inc. (Tokyo, Japan) for measurement of serum folate, homocysteine, and 25 OHVD levels. The serum folate level was measured by chemiluminescent enzyme immunoassay using the Access Folate Calibrators (Beckman Coulter, Inc., Tokyo, Japan) using Unicel^®^ DxI 800 (Beckman Coulter, Inc.). The serum homocysteine level was determined using high-performance liquid chromatography with fluorescence detection [26]. The 25 OHVD level was measured with double-antibody radioimmunoassay using the 25-Hydroxyvitamin D 125I RIA Kit (ScetiMedical Labo K.K., Tokyo, Japan) and Acetonitrile 300 (Wako Pure Chemical Industries Ltd., Osaka, Japan) as described previously [24] (reference range, folate level, >4.0 ng/mL; homocysteine level, 3.7–13.5 nmol/mL; 25 OHVD level, >30 ng/mL).

Oral mucosa samples were also obtained by sterilized cotton swab and sent to EBS Inc. (Hiroshima, Japan) for MTHFR genotype analysis. DNA was extracted from the oral mucosa using MagMAX™ DNA Multi-Sample Ultra Kit (Thermo Fisher Scientific, Tokyo, Japan) and KingFisher™ Flex Purification System (Thermo Fisher Scientific). Genotypes at C677T polymorphism in the *MTHFR* gene (rs1801133) were determined by a polymerase chain reaction method with confronting two-pair primers (PCR-CTPP) using KAPA2G™ Robust HotStart PCR kit (Nippon Genetics Co., Ltd., Tokyo, Japan). The sequence of primers was F1: 5′-CCA GCC TCT CCT GAC TGT CAT CC-3′, R1: 5′-AGC TGC GTG ATG ATG AAA TCG G-3′, F2: 5′-AGG AGA AGG TGT CTG CGG GAG T-3′, and R2: 5′-CCA TGT CGG TGC ATG CCT T-3′.

### 2.4. Statistical Analysis

The data were analyzed using the Kruskal–Wallis or chi-square test as appropriate. The Student’s *t*-test was used to compare the differences between pre- and post-multivitamin supplementation data. All statistical analyses were performed using GraphPad Prism version 6.07 for Windows (GraphPad Software, San Diego, CA, USA). Statistical significance was defined as a *p*-value < 0.05.

## 3. Results

### 3.1. Status of Folate, Homocysteine, Vitamin D and MTHFR Genotypes in Infertile Women

Regarding the distribution of MTHFR C677T, of the 205 women who participated in this study, 72 (35.1%) had wild-type CC, 100 (48.8%) had heterozygous mutant CT and 33 (16.1%) had homozygous mutant TT genotypes. Table 1 shows the characteristics of infertile patients in the three groups. The serum folate levels in the CC, CT and TT groups were 13.2 ± 6.5, 11.3 ± 5.5 and 9.2 ± 4.7 ng/mL, respectively (*p* = 0.002). Homozygous mutant, MTHFR 677TT genotype was associated with lower folate levels. The serum homocysteine levels in patients with a TT genotype (8.3 ± 3.9 nmol/mL) were significantly higher than those in patients with CC and CT genotypes (6.3 ± 1.4 and 6.6 ± 1.4 nmol/mL, respectively, *p* = 0.002). Three women (9.1%) with hyperhomocysteinemia were observed in only the TT group. Of patients with MTHFR 677CC, CT and TT, 71 (98.6%), 95 (95.0%) and 32 women (97.0%), respectively, had vitamin D insufficiency or deficiency. Most infertile women had 25 OHVD levels of <30 ng/mL; however, there was no significant difference in the three groups (*p* = 0.525). Of the 205 recruited women, 54 (26.3%) women had a risk of NTDs (folate level of <7.0 ng/mL and/or homocysteine level of >13.5 nmol/mL).

### 3.2. Impact of Multivitamin Supplementation on Folate and Homocysteine Levels

To identify the effects of multivitamin intervention on folate and homocysteine levels in infertile patients with a risk of NTDs, we examined the serum folate and homocysteine levels before and after multivitamin supplementation in 49 women with a folate level of <7.0 ng/mL and/or homocysteine level of >13.5 nmol/mL (Table 2). Multivitamin supplement intake for one month significantly increased the folate levels (5.8 ± 0.9 to 19.2 ± 4.0 ng/mL, *p* < 0.0001, Supplemental Figure S1A) and decreased the homocysteine levels (8.2 ± 3.1 to 5.8 ± 0.8 nmol/mL, *p* < 0.0001, Supplemental Figure S1B). The percentage change comparing the levels before and after multivitamin supplementation in the CC, CT and TT groups were +227.3%, +232.0%, and +246.3%, respectively, in the folate levels (*p* = 0.848) and −20.6%, −21.8%, and −33.5%, respectively, in the homocysteine levels (*p* = 0.175). Surprisingly, the serum folate levels in all patients with a risk of NTDs were increased to >7.0 ng/mL by multivitamin supplementation, and no women had hyperhomocysteinemia after one month.

### 3.3. Pregnancy Outcomes in Infertile Women with MTHFR 677CC, CT and TT

Of the 205 recruited women, 49 did not undergo fertility treatment because of the COVID-19 pandemic. Therefore, we examined pregnancy outcomes in 51, 79 and 26 women with MTHFR 677CC, CT and TT genotypes, respectively, within six months (Table 3). Sixty-eight (43.6%) and 88 (56.4%) women underwent intercourse or intrauterine insemination and assisted reproductive technology (ART) treatment, respectively. The total clinical pregnancy rates within six months in the CC, CT and TT groups were 45.1% (51 women), 39.2% (31 women) and 57.7% (15 women), respectively (*p* = 0.259). There were no significant differences in pregnancy rates after non-ART and ART treatments. We also compared the pregnancy outcomes in 42 and 114 women with and without multivitamin supplementation, respectively; there were no significant differences in cumulative pregnancy (21 women, 50.0% and 48 women, 42.1%, respectively, *p* = 0.468) and miscarriage rates (1 woman, 4.8% and 4 women, 8.3%, respectively, *p* = 1.000).

## 4. Discussion

This is the first study to identify the serum folate and homocysteine status and distribution of MTHFR C677T variations in infertile Japanese women. A previous study on MTHFR genotypes in 4517 Japanese individuals showed that 677CC, CT and TT were observed in 39.0%, 45.6% and 15.4%, respectively, which does not significantly differ from our data on infertile Japanese women [27]. According to previous studies, MTHFR genotype mutations are associated with miscarriage and RPL [16,28,29,30]. Moreover, MTHFR polymorphisms are also involved in poor ovarian stimulation responses in ART treatment [31], and folic acid supplementation improves ovulation disorders [32]. However, there were no significant differences in the miscarriage rate, length of menstruation cycle and rate of ovulation disorders in the three groups in our study. MTHFR gene mutations do not contribute to clinical pregnancy rates in ART treatment [33,34]; therefore, its polymorphisms may not cause infertility. However, multivitamin supplementation containing folic acid decreases the risk of pregnancy loss [35] and has a beneficial effect on female fecundity [21,32]. Furthermore, in the women with a history of RPL, MTHFR 677TT mutation is associated with vitamin D deficiency [16]. In our study with infertile women, there were no significant differences in the serum 25 OHVD level and the rates of vitamin D deficiency in all three groups. In all groups, ≥95% of women had vitamin D insufficiency or deficiency; thus, a significant difference might not be recognized.

When ≥7.0 ng/mL of the serum folate level is defined to minimize the risk of the incidence of NTDs based on a previous report [12], 26.3% (54/205) of infertile women require folic acid supplementation, including 12.5% (9/72 women), 30.0% (30/100 women) and 45.5% (15/33 women) of women with MTHFR 677CC, CT and TT, respectively. As the number of MTHFR mutation alleles increased, the number of women with serum folate <7 ng/mL increased, yet its proportion in women with homozygous mutant 677TT was less than half. Three quarters of the infertile women did not have NTD risk. Note that 9.1% of women with 677TT had hyperhomocysteinemia, but no women with 677CC and CT required multivitamin supplementation to prevent NTDs.

Regarding the effects of multivitamin intervention on folate and homocysteine levels depending on MTHFR genotypes, Hiraoka et al. reported that there were no significant differences of folate and homocysteine levels after 200 or 400 μg of folic acid supplementation among the 677CC, CT, and TT genotypes in women aged 36 to 81 years [36]. In our study, using multivitamins containing 800 μg of folic acid supplement for only one month increased the serum folate level to ≥7.0 ng/mL and decreased aberrant high homocysteine levels to a normal range. Regardless of MTHFR gene mutations, good improvement of folate and homocysteine levels in a shorter period than expected might result from multivitamin supplement use, Elevit^®^, containing not only folic acid but also vitamin B6 and B12. Furthermore, vitamin D supplement use also has an effect on inhibiting homocysteine [18,19]. In our study, the 25 OHVD level-dependent vitamin D supplementation in women without vitamin D sufficiency might support decreasing homocysteine levels. Regarding the pregnancy outcomes, there were no significant differences in clinical pregnancy and miscarriage rates in the three MTHFR genotype groups. Hyperhomocysteinemia, MTHFR 677TT mutation, and vitamin D deficiency are the risk factors for pregnancy loss [29,37,38,39]. Optimal multivitamin and additional vitamin D supplementation might contribute to pregnancy loss prevention after fertility treatments.

This study has some limitations. First, we had to stop this study and limit fertility treatment from 14 April 2020 to 13 May 2020, because of the COVID-19 pandemic. Therefore, we could not measure the folate and homocysteine levels in five women and could not follow the pregnancy outcomes in 49 women who did not visit our clinic. Second, the serum 25 OHVD levels were not confirmed after supplementation in this study. The vitamin B6 and B12 levels were also not measured. Lastly, there are no data on infants after fertility treatment with multivitamin supplement use because our hospital is a clinic specializing in fertility treatment.

## 5. Conclusions

A quarter of infertile (26.3%) women in this study had a serum folate concentration <7 ng/mL, a risk factor for NTDs. Over 95% of women had vitamin D insufficiency or deficiency. Regardless of MTHFR genotype variations, multivitamins in combination with additional vitamin D supplements were able to control the folate and homocysteine levels to minimize the risk of NTDs for one month. Tests for folate and homocysteine levels and optimal multivitamin supplementation in women with a risk of NTDs one month or more before pregnancy should be recommended to women who are planning a pregnancy as preconception care.

## Figures and Tables

**Table 1:** Characteristics of infertile women.

MTHFR C677T Genotypes	677CC (*n* = 72)	677CT (*n* = 100)	677TT (*n* = 33)	*p* Value
Age (years)	35.2 ± 3.9	35.3 ± 4.6	35.0 ± 4.4	0.923 ^b^
Duration of infertility (years)	1.6 ± 1.2	1.9 ± 1.8	1.7 ± 1.6	0.464 ^b^
History of pregnancy				
Gravida	0 (0–5)	0 (0–5)	0 (0–6)	0.207 ^b^
Para	0 (0–2)	0 (0–3)	0 (0–2)	0.459 ^b^
No. of previous miscarriages	0.4 ± 0.6	0.3 ± 0.6	0.3 ± 0.8	0.340 ^b^
Menstruation cycle (days)	31.8 ± 8.8	32.3 ± 9.0	33.0 ± 10.3	0.769 ^b^
Causes of infertility				
Tubal factors	8 (11.1)	7 (7.0)	3 (9.1)	0.600 ^c^
Endometriosis	4 (5.6)	5 (5.0)	2 (6.1)	1.000 ^c^
Ovarian factors	14 (19.4)	23 (23.0)	10 (30.3)	0.458 ^c^
(Ovulation disorders	10 (13.9)	14 (14.0)	7 (21.2)	0.564 ^c^
Male factors	7 (9.7)	15 (15.0)	3 (9.1)	0.574 ^c^
Unexplained	39 (54.2)	50 (50.0)	15 (45.5)	0.704 ^c^
Others ^a^	8 (11.1)	18 (18.0)	2 (6.1)	0.189 ^c^
Serum AMH (ng/mL)	3.7 ± 3.0	4.3 ± 4.8	3.9 ± 3.2	0.846 ^b^
Serum 25 OHVD (ng/mL)	14.8 ± 5.8	16.0 ± 7.7	17.3 ± 10.0	0.444 ^b^
Vitamin D insufficiency/defi ciency (<30 ng/mL)	71 (98.6)	95 (95.0)	32 (97.0)	0.525 ^c^
Serum folate (ng/mL)	13.2 ± 6.5	11.3 ± 5.5	9.2 ± 4.7	0.002 ^b^
≥7.0 ng/mL	63 (87.5)	70 (70.0)	18 (54.5)	<0.0001 ^c^
5.0−6.9 ng/mL	8 (11.1)	27 (27.0)	10 (30.3)	
3.0−4.9 ng/mL	1 (1.4)	3 (3.0)	5 (15.2)	
Serum homocysteine (nmol/mL)	6.3 ± 1.4	6.6 ± 1.4	8.3 ± 3.9	0.002 ^b^
Hyperhomocysteinemia (>13.5 nmol/mL)	0 (0)	0 (0)	3 (9.1)	0.004 ^c^

Values are average ± standard deviation or median (range) or *n* (%); AMH, anti-Müllerian hormone; 25 OHVD, 25-hydroxyvitamin D_3_. ^a^ Others causes of infertility include negative post-coital test outcome and uterine factor, such as endometrial polyps and submucosal myomas. ^b^ Student’s *t*-test. ^c^ Fisher’s extract test.

**Table 2:** Impact of multivitamin supplementation on folate and homocysteine levels.

MTHFR C677T Genotypes	677CC (*n* = 8)	677CT (*n* = 27)	677TT (*n* = 14)	Total (*n* = 49)	*p* Value ^b^
Folate levels					
Before supplementation (ng/mL)	5.8 ± 1.2	5.9 ± 0.7	5.7 ± 1.0	5.8 ± 0.9	0.935
After supplementation (ng/mL)	18.2 ± 5.9	19.3 ± 3.6	19.4 ± 3.8	19.2 ± 4.0	0.808
Percentage change ^a^ (%)	+227.3	+232.0	+246.3	+236.3	0.848
Homocysteine level					
Before supplementation (nmol/mL)	7.2 ± 1.3	7.4 ± 1.5	10.1 ± 5.0	8.2 ± 3.1	0.053
After supplementation (nmol/mL)	5.7 ± 1.1	5.7 ± 0.8	6.0 ± 0.8	5.8 ± 0.8	0.494
Percentage change ^a^ (%)	−20.6	−21.8	−33.5	−24.9	0.175

Values are average ± standard deviation. ^a^ Percentage changes of folate and homocysteine levels show mean fold changes comparing the levels before and after multivitamin supplementation. ^b^ *p* values were analyzed in the three groups of MTHFR 677CC, CT, and TT using the Kruskal–Wallis test.

**Table 3:** Pregnancy outcomes.

MTHFR C677T Genotypes	677CC (*n* = 51)	677CT (*n* = 79)	677TT (*n* = 26)	*p* Value
Age (years)	35.0 ± 3.7	35.0 ± 4.5	35.0 ± 4.8	0.998 ^a^
Pregnancy outcomes (<6 months)				
Intercourse or IUI	*n* = 25	*n* = 35	*n* = 8	
Cumulative preg- nancy rate	6 (24.0)	9 (25.7)	4 (50.0)	0.368 ^b^
Miscarriage rate	0 (0)	1 (11.1)	1 (25.0)	0.684 ^b^
IVF treatment	*n* = 26	*n* = 44	*n* = 18	
Cumulative preg- nancy rate	17 (65.4)	22 (50.0)	11 (61.1)	0.437 ^b^
Miscarriage rate	2 (11.8)	1 (4.5)	0 (0)	0.590 ^b^
Total	*n* = 51	*n* = 79	*n* = 26	
Cumulative preg- nancy rate	23 (45.1)	31 (39.2)	15 (57.7)	0.259 ^b^
Miscarriage rate	2 (8.7)	2 (6.5)	1 (6.7)	1.000 ^b^

Values are average ± standard deviation or *n* (%); IUI, intrauterine insemination; IVF, in vitro fertilization. ^a^ Student’s *t*-test. ^b^ Fisher’s extract test.

## Data Availability

The data that support the findings of this study are available on request from the corresponding author. The data are not publicly available due to privacy or ethical restrictions.

## Supplementary Materials

The following are available online at https://www.mdpi.com/article/10.3390/nu13041381/s1, Figure S1: Impact of multivitamin supplementation on folate and homocysteine levels. Serum folate (A) and homocysteine (B) levels before and after multivitamin supplementation for one month are shown in 49 women with folate level (*red dotted line*) of <7.0 ng/mL and/or homocysteine level (*red dotted line*) of >13.5 nmol/mL. All *P* values were analyzed using Student’s *t* test.
