# Supplementary material for: Effects of Periconceptional Multivitamin Supplementation on Folate and Homocysteine Levels Depending on Genetic Variants of Methyltetrahydrofolate Reductase in Infertile Japanese Women
Source: Nutrients. 2021 Apr 20;13(4):1381. doi: 10.3390/nu13041381 (PMC8073279; doi:10.3390/nu13041381)
Supplement: Supplementary file 1 [file nutrients-13-01381-s001.zip › nutrients-1129251-supplementary.pdf]

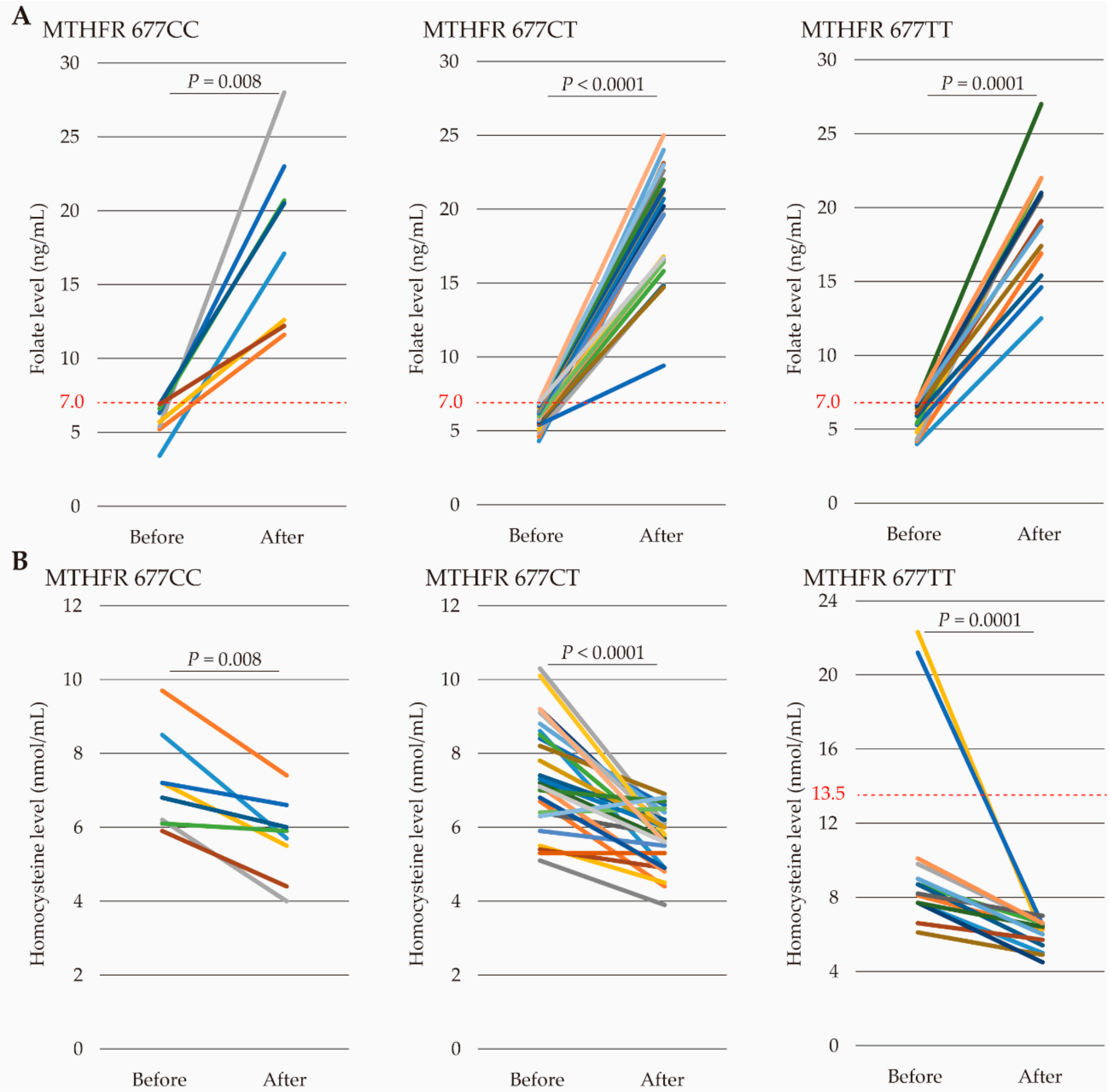

Supplemental Figure S1. Impact of multivitamin supplementation on folate and homocysteine levels. Serum folate (A) and homocysteine (B) levels before and after multivitamin supplementation for one month are shown in 49 women with folate level (*red dotted line*) of <7.0 ng/mL and/or homocysteine level (*red dotted line*) of >13.5 nmol/mL. All *P* values were analyzed using Student's t test.
